# METABOLOMIC SIGNATURES OF BRAIN ATROPHY PATTERNS IN AGING AND ALZHEIMER'S DISEASE

**DOI:** 10.1093/geroni/igac059.1233

**Published:** 2022-12-20

**Authors:** Qu Tian, Brendan Mitchell, Guray Erus, Christos Davatzikos, Susan Resnick, Luigi Ferrucci

**Affiliations:** National Institutes on Aging, Baltimore, Maryland, United States; National Institute on Aging, Baltimore, Maryland, United States; University of Pennsylvania, Philadelphia, Pennsylvania, United States; University of Pennsylvania, Philadelphia, Pennsylvania, United States; National Institute on Aging, Baltimore, Maryland, United States; National Institute on Aging, Baltimore, Maryland, United States

## Abstract

Can plasma metabolomics reveal mechanisms of brain aging? We investigated metabolomic signatures of brain atrophy patterns related to cognitive decline and Alzheimer’s disease(AD) risk. Relationships between metabolomics(Biocrates-p500) and annual rates of change in two neuroimaging-based brain atrophy patterns(SPARE-BA indexing brain aging, SPARE-AD indexing AD-related atrophy) were examined using multivariable linear regression in 477 Baltimore Longitudinal Study of Aging participants aged 60+, adjusted for demographic variables and BMI. Higher concentrations of sarcosine, triglycerides, diglycerides, and ceramides and lower concentrations from phosphatidylcholines and cholesteryl ester were associated with faster rates of SPARE-BA increase longitudinally. Higher concentrations of diglyceride and alpha-amino-butyric acid and lower concentrations of tryptophan, hippuric acid, cholesteryl ester, phosphatidylcholine and sphingomyelin were associated with faster rates of SPARE-AD. Metabolites have differential associations with age-related and AD-related brain atrophy patterns, which may provide new insights into preventive and therapeutic interventions. Future studies should examine whether metabolite changes precede brain atrophy patterns.

